# Automated dynamic phenotyping of whole oilseed rape (*Brassica napus*) plants from images collected under controlled conditions

**DOI:** 10.3389/fpls.2025.1443882

**Published:** 2025-05-22

**Authors:** Evangeline Corcoran, Kasra Hosseini, Laura Siles, Smita Kurup, Sebastian Ahnert

**Affiliations:** ^1^ The Alan Turing Institute, London, United Kingdom; ^2^ Zalando Societas Europaea (SE), Berlin, Germany; ^3^ Department of Plant Science and the Bioeconomy, Rothamsted Research, Harpenden, United Kingdom; ^4^ Department of Chemical Engineering and Biotechnology, University of Cambridge, Cambridge, United Kingdom

**Keywords:** plant phenotyping, computer vision, machine learning, image analysis, plant development

## Abstract

**Introduction:**

Recent advancements in sensor technologies have enabled collection of many large, high-resolution plant images datasets that could be used to non-destructively explore the relationships between genetics, environment and management factors on phenotype or the physical traits exhibited by plants. The phenotype data captured in these datasets could then be integrated into models of plant development and crop yield to more accurately predict how plants may grow as a result of changing management practices and climate conditions, better ensuring future food security. However, automated methods capable of reliably and efficiently extracting meaningful measurements of individual plant components (e.g. leaves, flowers, pods) from imagery of whole plants are currently lacking. In this study, we explore interdisciplinary application of MapReader, a computer vision pipeline for annotating and classifying patches of larger images that was originally developed for semantic exploration of historical maps, to time-series images of whole oilseed rape (*Brassica napus*) plants.

**Methods:**

Models were trained to classify five plant structures in patches derived from whole plant images (branches, leaves, pods, flower buds and flowers), as well as background patches. Three modelling methods are compared: (i) 6-label multi-class classification, (ii) a chain of binary classifiers approach, and (iii) an approach combining binary classification of plant and background patches, followed by 5-label multi-class classification of plant structures.

**Results:**

A combined plant/background binarization and 5-label multi-class modelling approach using a ‘resnext50d_4s2x40d’ model architecture for both the binary classification and multi-class classification components was found to produce the most accurate patch classification for whole *B. napus* plant images (macro-averaged F1-score = 88.50, weighted average F1-score = 97.71). This combined binary and 5-label multi-class classification approach demonstrate similar performance to the top-performing MapReader ‘railspace’ classification model.

**Discussion:**

This highlights the potential applicability of the MapReader model framework to images data from across scientific and humanities domains, and the flexibility it provides in creating pipelines with different modelling approaches. The pipeline for dynamic plant phenotyping from whole plant images developed in this study could potentially be applied to imagery from varied laboratory conditions, and to images datasets of other plants of both agricultural and conservation concern.

## 1 Introduction

The phenotype, or observable and quantifiable physical traits of individual plants, represents the interaction between their genetics, environment, and management conditions (Costa et al., 2019; Walter et al., 2015). The collection of accurate phenotype data is therefore essential for improving our understanding of the fundamental developmental mechanisms in plants and for the future of plant breeding, as this will allow for identification of advantageous genes and management practices to ensure plants are optimally suited for growth and production of high yields in their given environment (Costa et al., 2019; Walter et al., 2015). As climate conditions are predicted to continue to change rapidly over the coming decades, leading to novel or intensified environmental pressures on agricultural crops, accurate and rapid plant phenotyping are becoming increasingly integral to ensuring future food security (Campbell et al., 2016; Corcoran et al., 2023a; Lobell et al., 2011; Maraveas, 2024). However, conventional manual methods for collecting plant phenotype data are often time and labor intensive, and often require destruction of the plants being measured (Campbell et al., 2016; Lobell et al., 2011). Therefore phenotype data cannot be collected with the frequency required to gain an in-depth understanding of the dynamics of plant growth, including how development of different parts of plants may affect each other throughout their growth cycle, or how fine temporal resolution changes may be used to predict yield or abnormal development (Corcoran et al., 2023b; Costa et al., 2019).

Sensor-based automated phenotyping can capture plant growth dynamics more efficiently, however, the precision of image segmentation tools remains a persistent challenge. Although advancements in image systems and software have improved mask creation for more accurate plant segmentation, these tools often require manual adjustments, complicating the separation of individual plant organs. Automated methods using machine learning-based computer vision models have the potential to significantly improve the accuracy and efficiency of data extraction from images of crops at various scales (Gill et al., 2022; Li et al., 2020). These methods have been primarily developed for analysis on two kinds of plant imagery; plant organ images and images of whole plants (Das Choudhury et al., 2019; Gill et al., 2022; Li et al., 2020). Application of machine learning-based object detection and segmentation models to count or collect measurements of a specific plant organ such as leaves (Aich and Stavness, 2017; An et al., 2017; Dobrescu et al., 2017; Golbach et al., 2016; Lee et al., 2020; Namin et al., 2018), roots (Wan et al., 2019; Xu et al., 2018), and seeds (Corcoran et al., 2023b) have demonstrated high accuracy and efficiency. However, automated analysis of whole individual plants has proven more challenging (Gill et al., 2022; Li et al., 2020). This is due to the complexity of whole plants as they are made up of multiple connected organs that can be hard to segment and often overlap or occlude each other in two-dimensional images (Gill et al., 2022; Li et al., 2020). Use of multiple angles for 2D images, as well as 3D imaging, have been proposed as viable solutions to inaccuracies introduced by overlapping plant parts (An et al., 2017; Das Choudhary et al., 2020; Gelard et al., 2017; Harandi et al., 2023; Paulus, 2019; Xu et al., 2019). However, 3D images tend to be large and computationally expensive to analyze, so this type of analysis may not be practical when the aim of research is to extract data on the overall abundance and distribution of various plant organs (e.g. leaves, flowers, pods) and how these change across a time-series of images (Gelard et al., 2017; Harandi et al., 2023; Paulus, 2019; Xu et al., 2019).

MapReader is an openly accessible and open-source Python software library that allows users with limited expertise in computer vision to load image data and divide original images into patches which can be manually annotated to train deep convolutional neural network models to classify patches in novel images (Hosseini et al., 2022). MapReader was originally developed for semantic exploration of historical maps at scale, specifically to classify railways and related infrastructure (‘railspace’) in maps and explore how railspace changed over time and how it related to other structures around it (Hosseini et al., 2022). Through the ‘railspace’ use case it was demonstrated how the abundance and distribution of a branching structure (railway lines), and connected or adjacent related structures (railway-related buildings) could be accurately classified within complex two-dimensional images, and compared across time-series images (Hosseini et al., 2022). Therefore, this method appeared promising for application to analysis of whole plant images as a MapReader model could be trained on patches taken from these images and could allow for computationally efficient segmentation of branches (a structure similar in appearance in 2D images to rail networks) and connected plant organs (Hosseini et al., 2022). In particular, the MapReader pipeline offered a potential advantage in that spatial relationships between patches could be used to better inform patch classification and produce more accurate predictions of plant organ abundance and distribution (Hosseini et al., 2022).

This paper explores the application of the MapReader pipeline to the extraction of data on plant phenotypes from images of whole oilseed rape (*Brassica napus*, hereinafter *B. napus*) plants (Hosseini et al., 2022; Wood et al., 2024). The performance of multi-class models for classification of six classes of patch (background, branch, leaf, flower bud, flower and pod), an approach involving a chain of binary classifier models for each of the aforementioned classes, and a combined approach where the image is first classified into plant and non-plant patches, with plant patches then being further classified into the five plant organ classes (branch, leaf, flower bud, flower, pod) are compared in terms of accuracy and efficiency. The capacity to examine growth trends by comparing classification of images of individual whole plants across time-series is then demonstrated, and the potential use of data extracted using the adapted MapReader pipeline to improve models of plant development is discussed.

## 2 Materials and methods

### 2.1 Data collection and annotation

**Figure 1:**
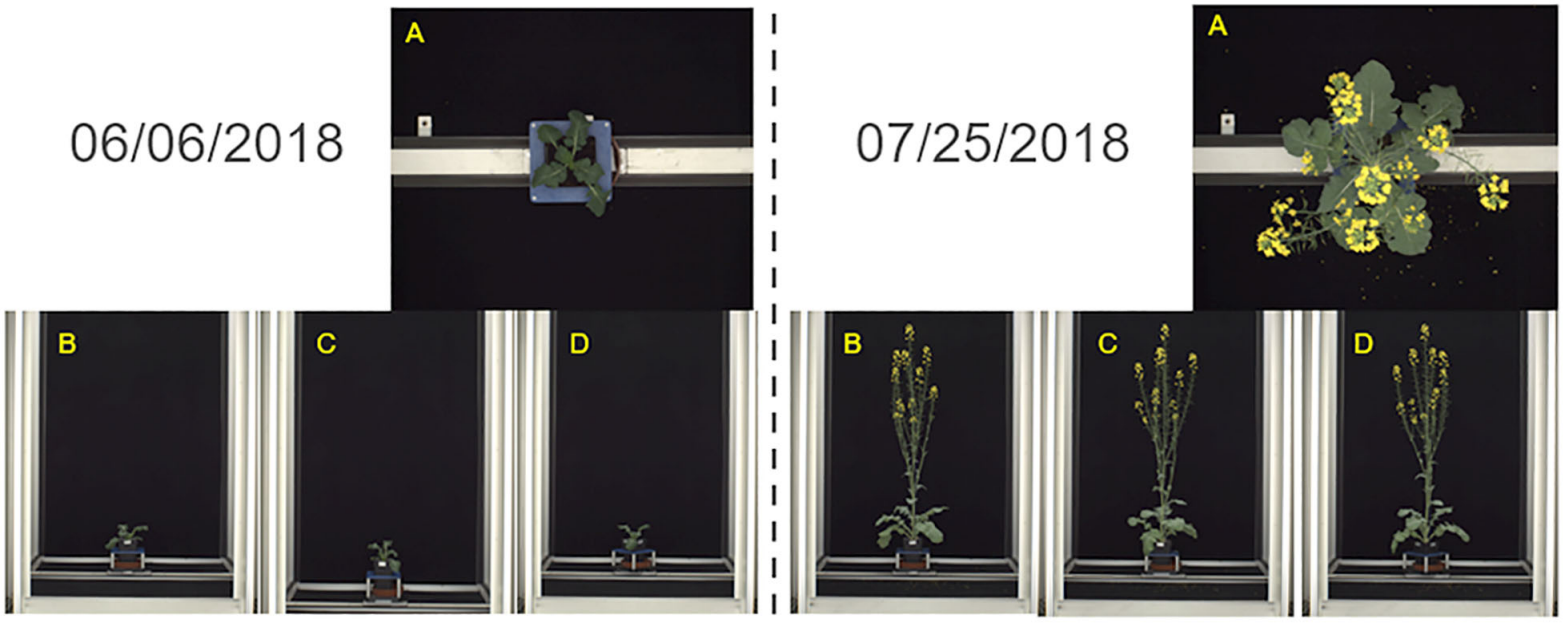
Images of whole *Brassica napus* plants collected by Williams et al. (2023a) using the LemnaTec Scanalyzer system used to develop the plant patch classification model. Images were collected daily for six weeks from 06/06/2018-07/25/2018 from a top-down view **(A)**, and side views at 0- **(B)**, 45- **(C)**, and 90- **(D)** degree angles.

A sub-set of 384 color (RGB) images of three whole *Brassica napus* plants from the ‘Collection of side view and top view RGB images of *B. napus* from a large scale, high throughput experiment’ created by Williams et al. (2023a) were used in the development of the plant patch classification model. These images were originally collected as part of an experiment to assess flowering response in *B. napus* in response to varying levels of winter cold, or vernalization (Williams et al., 2023b). All plants were grown in an environmentally controlled glasshouse and images were collected daily for six weeks from 6th June 2018 to 25th July 2018 (Williams et al., 2023a). Plants were imaged using a semi-automated conveyor system (LemnaTec Scanalyzer) which transported plants to a photo booth in which 4 RGB images were collected per day, comprising of 1 top-down view image and 3 side view images at 0-, 45-, and 90-degrees rotation (Williams et al., 2023a) (**Figure 1**). All top-down view images were consistent in scale with each other and shared a 2454 by 2056 pixels resolution. Side-view images were all consistent in scale with each other regardless of the degree of plant rotation and shared a 2056 by 2454 pixels resolution. Metadata attached to each image identified the individual *B. napus* plant, the data of image capture, genotype information, and which of two experimental vernalization treatments the plant was exposed to prior to flowering (Williams et al., 2023b). Side-view images from all angles were selected to perform model training, validation, and testing due to the lack of occlusion of different plant parts compared to the top-down view images.

**Figure 2:**
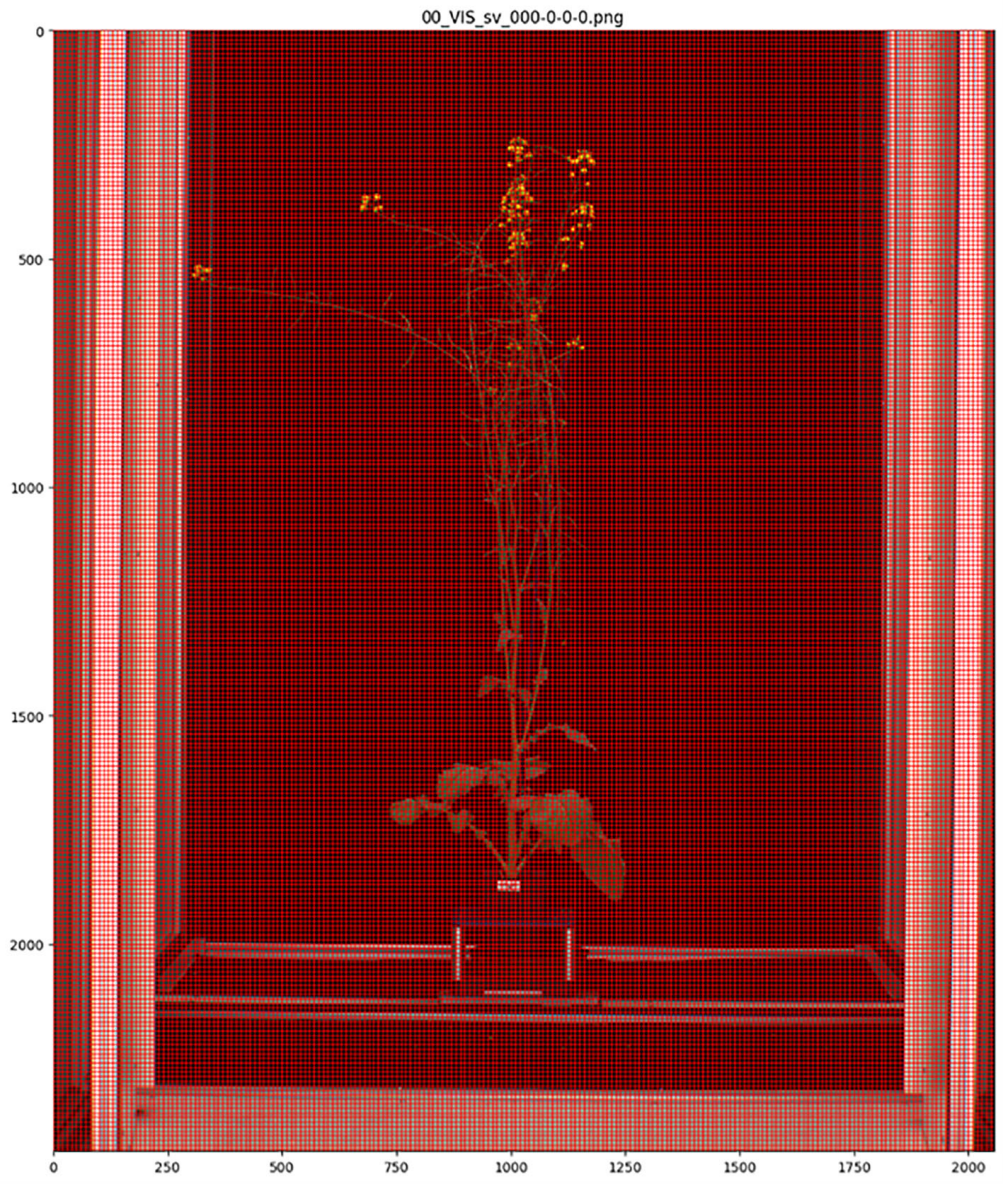
Side-view image of whole *Brassica napus* plant divided into 10 x 10-pixel patches using the MapReader toolkit.

**Figure 3:**
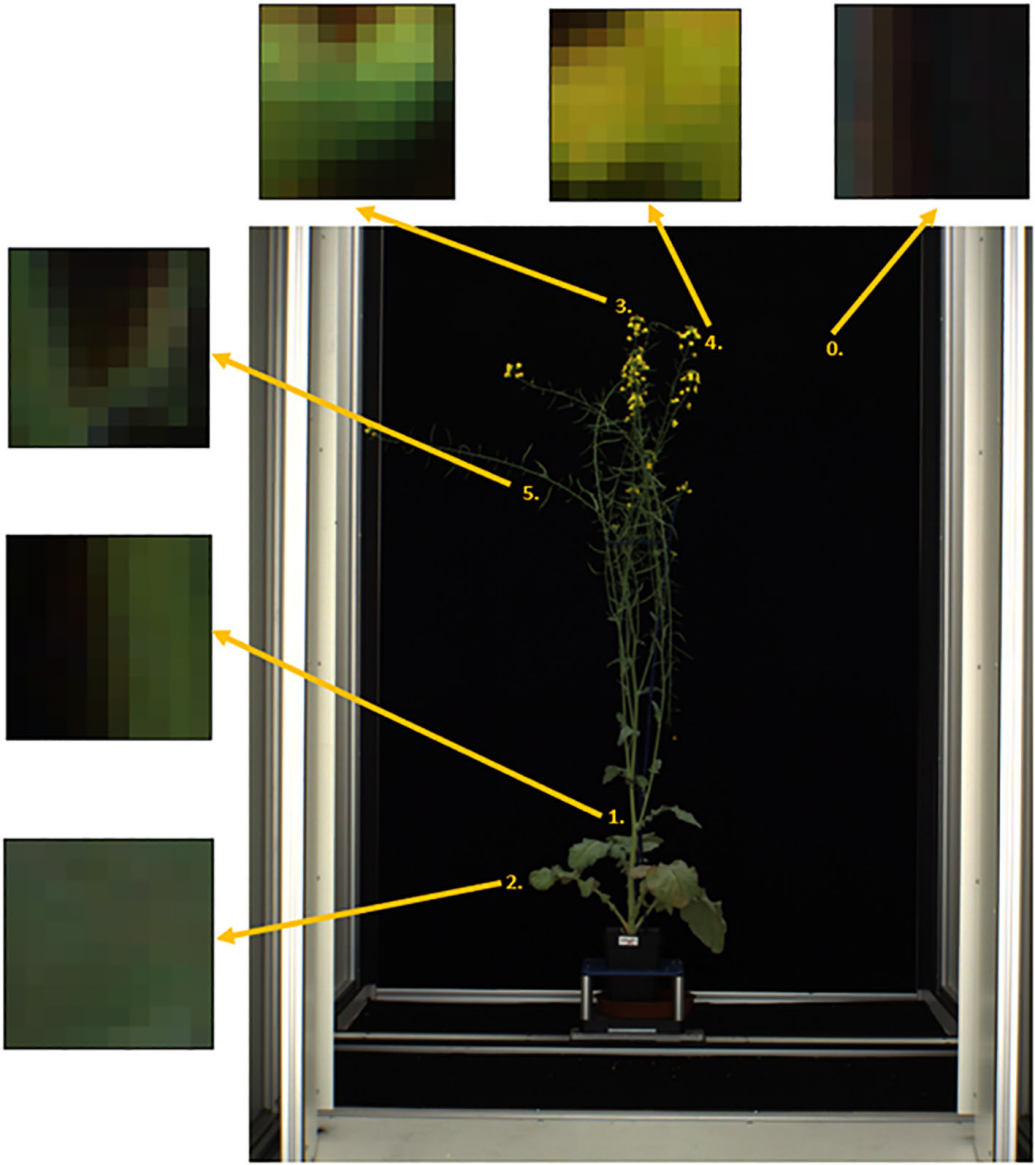
Labelled patches for each class derived from side-view images of whole *Brassica napus* plants including 5 plant parts (1 = ‘branch’, 2 = ‘leaf’, 3 = ‘bud’, 4 = ‘flower’, 5 = ‘pod’) and image background (0).

MapReader version 0.3.3 was used to carry out image annotation, model training and model inference (https://github.com/Living-with-machines/MapReader). Each side-view whole plant image was sliced into a grid of 10 x 10-pixel patches using the MapReader toolkit (Hosseini et al., 2022) (**Figure 2**). This patch size was selected as it was the largest patch size for which the majority of patches contained only a single class of plant part, as the MapReader pipeline only allowed providing each patch with a single label. Patches were then labelled using the MapReader annotation interface, which allowed the annotator to view each patch in isolation and assign it a label based on the predominant plant organ or structure present within the patch (Hosseini et al., 2022). These labels included five classes for different plant organs and structures (branches = ‘branch’, leaves = ‘leaf’, flower buds = ‘bud’, open flowers = ‘flower’ and green pods containing seeds = ‘pod’) and one class for patches that did not contain any part of a plant (‘background’) as shown in **Figure 3**; **Supplementary Image 1-6**. As the flower buds, mature flowers, and pods tended to appear only later in the growth period of each plant, the majority of annotated patches were taken from images collected after 1^st^ July 2018. After all, ‘background’, ‘leaf’ and ‘branch’ patches were annotated, remaining patches were filtered based on pixel color in order to retrieve supplemental ‘flower’, ‘bud’ and ‘pod’ patches as these were smaller and present in less images than other classes, therefore patches containing these plant structures were rarer.

The total number of patches annotated was 62,303 (**Supplementary Table 1**). Of these, the majority contained ‘background’ (44,872), with 7,942 ‘leaf’ patches, 5,426 ‘branch’ patches, 2,488 ‘flower’ patches, 839 ‘pod’ patches and 736 ‘bud’ patches (**Supplementary Table 1**). Due to the highly unbalanced nature of the dataset, a stratified sampler was used to split the total dataset into training, validation and testing batches, allocating 60%, 20% and 20%, respectively. To train models a weighted sampler was used to ensure that each classifier model was shown examples from all classes, including those that were underrepresented in the dataset. The sample of patches shown to classifier models during training was defined where weight for each class was calculated as:


$$class\;weight=\frac{1}{\left(\frac{\left(class\;sample\;count\right)}{10}\right)}$$

This resulted in the following weights for each class: ‘background’ = 0.0004, ‘flower’ = 0.0067, ‘bud’ = 0.0226, ‘leaf’ = 0.0021, ‘pod’ = 0.0199, and ‘branch’ = 0.0031. This resulted in a model training dataset comprised of 37,381 patches, a validation dataset comprised of 12,461 patches and a testing dataset also comprised of 12,461 distinct patches (**Supplementary Table 1**).

In this case, it was found not to be necessary to apply an further preprocessing steps to patches before they were used in model training and inference, although the MapReader pipeline does allow patches to be normalized based on the mean and standard deviations of pixel intensities where relevant (Hosseini et al., 2022).

### 2.2 Model training, validation and testing

The following model architectures that were previously trained to detected ‘railspace’ as demonstrated by Hosseini et al., 2022 were fine-tuned using the training dataset for classification of patches derived from images of whole *B. napu*s plants; ‘resnest101e’ with and without generalized pre-training (Zhang et al., 2020), ‘resnext50d_4s2x40d’ (Yalniz et al., 2019), ‘tf_efficientnet_b3_ns’ (Tan and Le, 2020), ‘resnet152’ (He et al., 2015), ‘resnext101_32x8d’ (Yalniz et al., 2019), ‘vit_base_patch32_224’ (Dosovitskiy et al., 2020), and ‘swin_base_patch4_window7_224_in22k’ (Liu et al., 2021). These model architectures fine-tuned on ‘railspace’ data can be accessed for further fine-tuning on other annotated datasets at https://huggingface.co/papers/2111.15592.

Three modelling approaches for classification of whole *B. napus* plant image patches were explored. Firstly, a 6-label multi-class modelling approach in which all model architectures were fine-tuned using the entire training dataset including labelled patches of all five plant organ classes (‘branch’, ‘leaf’, ‘bud’, ‘flower’ and ‘pod’) and labelled background patches. These were then validated using the entire validation dataset, with the output of these models being a prediction of which of the six total classes each patch was most likely to contain, and the confidence level for that classification.

The second classification method explored was a chain of binary-classifiers approach. For this approach, a binary-classifier model with each model architecture was fine-tuned for each of the six classes (‘flower’, bud’, ‘leaf’, ‘branch’, ‘pod’, and ‘background’) by adjusting the labels of the annotated patches so that a label of ‘1’ indicated that the patch contained the relevant class, and a label ‘0’ indicated that it did not contain the relevant class. So, for example, when training a binary classifier for classification of ‘flower’ patches a label of ‘1’ indicated that the patches contained flowers, and ‘0’ indicated that the patch contained another plant organ or background. The output of these models was a prediction label of ‘0’ or ‘1’ for each class for each patch, as well as a confidence value. The results of binary-classifiers for all six classes could then be combined by taking the class with a ‘1’ label and highest confidence value as the final prediction for each patch.

The final modelling approach explored was a two-step process combining a binary-classifier and multiclass model. The first step involved classifying all patches into either ‘background’ or ‘plant’ patches. To achieve this, the top-performing binary-classifier model for ‘background’ patches developed for the second approach was used, therefore this step did not require additional model training and validation. For the second step, a 5-label multi-class model was trained with each model architecture to classify patches into the five plant organ classes (‘flower’, bud’, ‘leaf’, ‘branch’, and ‘pod’). Only patches containing plants, meaning only patches assigned a ‘0’ label by the top-performing ‘background’ binary-classifier model used in the first step, were passed to the second 5-label plant classification step. Therefore ‘background’ patches were filtered out and the 5-label multi-class models were therefore trained on a subset of 10,458 patches, validated on a subset of 3,486 patches, and test on a subset of 3,486 patches from the original training dataset that contained plant parts (**Supplementary Table 2**).

For each of the three classification approaches outlined above, all model architectures were trained, tested and validated using a Baskerville Tier 2 high-performance computing service (HPC) compute node with 512GB RAM, an Intel^®^ Xeon^®^ Platinum 8360Y CPU with 36 cores at 2.4GHz (with boost to 3.5GHz) and a single NVIDIA A100 40GB graphics processing unit (GPU). The training patch size was 10 by 10 pixels equal to the size of patches sliced from the original *B. napus* images, the batch size was 16, and training ran for 50 epochs. The learning rate for all model architectures was set to 0.001, and an ‘Adam’ optimization algorithm was used for all models (Kingma and Ba, 2017). The scheduler parameters for all model architectures was step size = 10, gamma = 0.1 and last epoch = -1. Training took on average 125 minutes to complete for each model and models were trained concurrently on the Baskerville Tier 2 HPC service.

Accuracy of patch classification for the validation and testing datasets was measured using the recall, precision, and F1-score. Recall values were calculated as:


$$Recall=\frac{TP}{TP+FN}$$

Where *TP* equaled ‘true positive results,’ or the number of patches from each class that were correctly assigned that class label during model inference, and *FN* equaled ‘false negative results’, or the number of patches from each class that were incorrectly assigned another label that did not match the one assigned during manual annotation.

Precision values were calculated as:


$$Precision=\frac{TP}{TP+FP}$$

Where *TP* equaled the number true positive results as above, and *FP* equaled the number of patches that were incorrectly classified as belonging to each class.

F1-score (*F*) was then calculated as:


$$F=2\cdot \frac{Precision\cdot Recall}{Precision+Recall}$$

For each of these measures (recall, precision, and F1-score) values were reported using three aggregation methods, resulting in a micro-averaged, macro-average, and weighted average score for each metric. Micro-averaged values were calculated using the combined total number of true positive (TP), false negative (FN), and false positives (FP) without considering the proportion of labels in the dataset. For macro-averaged values recall, precision and F1-score were first calculated for each class, and then averaged by the number of classes without considering the proportion of labels in each class. Weighted average values were calculated by first calculating recall, precision, and F1-score for each class and then returning the average considering the proportion of labels of each class in the dataset.

Although the dataset was highly unbalanced with 72% of labelled patches being ‘background’, reliable classification of the five plant classes (‘flower’, ‘pod’, ‘leaf’, ‘bud’, and ‘branch’) was considered high priority, as this was determined to be critical to allowing accurate assessment the rate and pattern of growth of these plant structures over time from the whole plant images. Therefore, it was decided model performance would be ranked by macro-averaged F1-score to emphasize accurate classification of minority plant structure classes, rather than by micro-averaged or weighted F1-score measures that were more representative of accuracy in classification of background versus plant patches.

## 3 Results

### 3.1 Model performance on validation dataset

#### 3.1.1 6-label multi-class models

For the validation dataset, the top performing six label multi-class model for classification of different *B. napus* plant structures was a ‘resnest101e’ neural network (**Table 1**). This unweighted macro-average accuracy of this model across all classes was 89.06% and the micro-average 97.89%. When averaged across performance on all classes, and weighted for class imbalance in the validation dataset, the overall accuracy of the ‘resnest101e’ model was 97.89%. The macro-average recall across all classes was 88.73%, meaning that the mean rate of false negative errors across classes was 11.27%. When weighted based on the imbalance number of examples of each class in the validation dataset, the average recall was 97.89%, indicating a weighted false negative rate of 2.11%. The macro-average precision was 89.46, indicating a 10.54% false positive rate when averaged across all classes. This was lower than the 90.07% macro-average precision of the ‘resnest101e_no_pretrain’ which performed second best on the validation dataset when ranked according to weighted F1 score. However, the weighted precision of the ‘resnest101e’ model (97.89%) was higher than that of the ‘resnest101_no_pretrain’ model (97.76%) when accounting for class imbalance. As shown in **Table 1** the ‘resnest101e’ model was the top performing model for classification of patches containing five out of six individual classes: ‘background’, ‘bud’, ‘leaf’, ‘pod’, and ‘branch’. Of these five classes this model most accurately differentiated ‘background’ patches from patches containing plant parts (F1-0 = 99.87%). ‘Flower,’ ‘leaf’, and ‘branch’ patches were also classified with over 90% accuracy using this model architecture, while classification of ‘bud’ (F1-2 = 76.25%) and ‘pod’ (F1-4 = 76.14) patches was comparatively less accurate. For classification of ‘flower’ patches, the top performing model architecture was ‘resnext50d_4s2x40d’ with the overall top performing model, ‘resnest101e’, ranking third behind ‘resnest101e_no_pretrain’. Classification of all patches included in the validation dataset was completed using a single GPU in 46 seconds. Micro- and weighted average F1-score for all 6-label multiclass models on the training dataset can be found in **Supplementary Table 3**.

**Table 1:** Performance of 6-label multi-class computer vision classifier models on the validation set with 12,461 labelled patches (20% of all manually annotated patches) including macro-averaged recall and precision in descending order according to F1-macro.

Model Name	Rec-macro	Prec-macro	F1-macro	F1-0	F1-1	F1-2	F1-3	F1-4	F1-5	Time
‘resnest101e’	**88.73**	89.46	**89.06**	**99.87**	**91.90**	**96.39**	**76.25**	93.78	**76.16**	0m 46s
‘vit_base_patch32_224’	88.16	83.43	85.36	99.77	88.31	94.91	69.23	94.09	65.85	0m 24s
‘resnext101_32x8d’	85.03	86.87	85.91	**99.87**	88.14	94.67	70.59	91.19	71.03	0m 40s
‘resnet152’	84.73	88.37	86.13	99.82	88.55	94.68	71.49	93.81	68.47	0m 34s
‘tf_efficientnet_b3_ns’	85.62	87.31	86.32	99.83	90.28	95.50	70.07	92.93	69.31	0m 28s
‘resnext50d_4s2x40d’	86.17	88.97	87.41	**99.87**	90.53	95.42	71.85	**94.22**	72.56	0m 48s
‘resnest101e_no_pretrain’	87.18	**90.07**	88.41	99.80	91.67	96.12	74.05	94.00	74.85	0m 46s
‘swin_base_patch4_window7_224_in22k’	16.67	0.20	0.39	0.0	0.0	0.0	2.33	0.0	0.0	0m 45s

The F1-score for each class label is also listed. F1-0: ‘Background’; F1-1: ‘Branch’; F1-2: ‘Leaf’; F1-3: ‘Bud’; F1-4: ‘Flower’; and F1-5: ‘Pod’. Time is for model inference on the total 12,461 patch dataset. Bold values indicate the top-performing model for each performance metric specified by column name.

#### 3.1.2 Chain of binary classifier models

The overall top performing model architecture for classification of *B. napus* plant parts in the validation dataset was ‘resnext50d_4s2x40d’ with a macro-averaged F1-score of 88.58% and a weighted average F1-score across all six classes of 98.08% (**Table 2**). As shown in **Table 2**, the ‘resnext50d_4s2x40d’ model demonstrated the highest accuracy for five out of six individual classes (‘background’, ‘flower’, ‘bud’, ‘leaf’, and ‘branch’) compared to other model architectures. Binary classification using the ‘resnext50d_4s2x40d’ model was most accurate for differentiating ‘background’ patches from plant parts and was similar to the accuracy of background classification using the top performing multi-class model. Classification of ‘flower’, ‘leaf’, and ‘branch’ patches was marginally more accurate and classification of ‘bud’ patches was marginally less accurate using the chain of binary classifier approach compared to the top performing multi-class model, with the difference in accuracy for classification of all aforementioned classes between the top performing multi-class and binary classifier models being less than 2% (**Tables 1**, **2**). Similar to the top performing multi-class model, the ‘pod’ class was classified with the lowest accuracy. There was also the largest discrepancy in accuracy between model approaches for ‘pod’ patches, with the binary classifier model demonstrating 4.35% lower accuracy than the top performing multi-class model (**Tables 1**, **2**). Additionally, ‘resnext50d_4s2x40d’ was not the highest performing model architecture for binary ‘pod’ patch classification as a ‘resnest101e_no_pretrain’ binary classifier model demonstrated 74.21% accuracy on the validation dataset, though this was still lower than the accuracy of ‘pod’ classification for the top performing multi-class model (**Tables 1**, **2**). The total time taken to complete binary classification of the validation dataset with the ‘resnext50d_4s2x40d’ model was 5 minutes 14 seconds with a single GPU, however it should be noted that with access to a GPU cluster capable of running multiple jobs, binary classification for each class could be run simultaneously reducing the time required to 52 seconds (**Table 2**). Weighted average F1-score for all binary classifier models on the training dataset can be found in **Supplementary Table 4**.

**Table 2:** Performance of computer vision binary classifier models on the validation set with 12,461 labelled patches (20% of all manually annotated patches) in descending order according to F1-macro. The F1-score for each class label is also listed.

Model Name	F1-macro	F1-0	F1-1	F1-2	F1-3	F1-4	F1-5	Time
‘resnext50d_4s2x40d’	**88.58**	**99.88**	**92.36**	**96.75**	**75.80**	**94.89**	71.81	5m 14s(0m 52s)
‘resnest101e_no_pretrain’	87.50	99.85	91.04	95.54	71.34	92.99	**74.21**	4m 50s(0m 49s)
‘resnest101e’	87.29	99.81	89.83	95.98	72.37	94.43	71.34	4m 48s(0m 48s)
‘tf_efficientnet_b3_ns’	87.15	99.85	91.46	95.21	74.26	92.57	69.54	2m 42s(0m 27s)
‘resnet152’	87.09	99.15	90.24	94.69	73.76	93.81	70.87	3m 13s(0m 32s)
‘resnext101_32x8d’	85.82	99.70	89.76	95.28	67.34	93.70	69.14	3m 51s(0m 39s)
‘vit_base_patch32_224’	73.33	99.70	82.87	92.84	24.14	93.89	46.55	2m 33s(0m 26s)
‘swin_base_patch4_window7_224_in22k’	18.17	83.73	0.0	22.61	0.0	0.0	2.66	4m 12s(0m 42s)

F1-0, ‘Background’; F1-1, ‘Branch’; F1-2, ‘Leaf’; F1-3, ‘Bud’; F1-4, ‘Flower’; and F1-5, ‘Pod’. Time is for model inference on the total 12,461 patch dataset. Bold values indicate the top-performing model for each performance metric specified by column name.

#### 3.1.3 Combined plant/background binarization and 5-label multi-class model of plant structures

The top-performing 5-label (‘flower’, ‘bud’, ‘leaf’, ‘pod’, ‘branch’) model architecture for classifying *B. napus* plant structures in the validation dataset following binary classification of plant patches from background patches based on macro-averaged F1-score was ‘resnext50d_4s2x40d’ (F1-macro = 86.22%) (**Table 3**). However, the top performing model architecture when accounting for class imbalance was ‘resnest101e_no_pretrain’ with a weighted F1-score of 91.91%. The ‘resnest101e_no_pretrain’ model architecture performed best at classification of ‘leaf’, ‘pod’ and ‘branch’ patches while the ‘resnext50d_4s2x40d’ architecture performed best at classification of ‘bud’ patches. A ‘resnest101e’ model performed best at classification of ‘flower’ patches and ranked second best in terms of weighted F1-score and fourth by macro-average F1-score. After combining the results of binary plant/background classification with 5-label classification of plant structures, the top-performing model architecture was ‘resnext50d_4s2x40d’ with a macro-average F1-score of 88.50% (**Table 4**). Classification of plant patches in the validation dataset using the overall top performing model architecture (‘resnest101e_no_pretrain’) took 14 seconds to complete using a single GPU (**Table 3**). Micro- and weighted average F1-score for these models on the training dataset can be found in **Supplementary Tables 5**, **6**.

**Table 3:** Performance of 5-label multi-class computer vision classifier models on the validation set with 12,461 labelled patches (20% of all manually annotated patches) in descending order according to F1-macro.

Model Name	Rec-macro	Prec-macro	F1-macro	F1-1	F1-2	F1-3	F1-4	F1-5	Time (m)
‘resnext50d_4s2x40d’	87.28	85.30	**86.22**	89.93	95.51	**77.52**	94.22	73.92	0m 14s
‘resnext101_32x8d’	85.22	**86.44**	85.52	90.11	95.41	73.49	93.62	75.0	0m 14s
‘tf_efficientnet_b3_ns’	86.48	84.58	85.42	90.73	95.32	74.58	93.59	72.88	0m 9s
‘resnest101e’	85.87	84.78	85.32	90.50	95.53	74.74	**94.47**	71.35	0m 14s
‘vit_base_patch32_224’	**88.77**	85.85	85.21	89.20	94.92	72.20	94.28	75.44	0m 7s
‘resnest101e_no_pretrain’	85.76	84.45	84.96	**90.93**	**95.82**	68.10	93.93	**76.03**	0m 14s
‘resnet152’	85.48	84.90	84.69	89.28	95.30	72.45	94.33	72.08	0m 9s
‘swin_base_patch4_window7_224_in22k’	20.0	9.11	12.52	0.0	62.59	0.0	0.0	0.0	0m 12s

The F1-score for each class label is also listed. F1-0, ‘Background’; F1-1, ‘Branch’; F1-2, ‘Leaf’; F1-3, ‘Bud’; F1-4, ‘Flower’; and F1-5, ‘Pod’. Time is for model inference on the total 12,461 patch dataset. Bold values indicate the top-performing model for each performance metric specified by column name.`

**Table 4:** F1-scores for combined results of plant/background classification using the top performing binary classifier model (‘resnext50d_4s2x40d’, see **Table 2**) and 5-label multiclass plant patch classification (**Table 3**) for the validation dataset.

Model Name	F1-macro
‘resnext50d_4s2x40d’	**88.50**
‘resnext101_32x8d’	87.91
‘tf_efficientnet_b3_ns’	87.83
‘resnest101e’	87.75
‘vit_base_patch32_224’	87.65
‘resnest101e_no_pretrain’	87.44
‘resnet152’	87.22
‘swin_base_patch4_window7_224_in22k’	27.08

Bold values indicate the top-performing model for each performance metric specified by column name.

### 3.2 Model performance on test dataset

#### 3.2.1 6-label multi-class models

The top performing 6-label multiclass model for classifying *B. napus* plant structures in the test dataset was ‘resnext101_32x8d’ with a macro-average F1-score of 90.28%, which also provided the most accurate classification of ‘flower’ and ‘branch’ patches for the validation dataset (**Table 5**). When accounting for class imbalance, the top-preforming model architecture was ‘resnest101e_no_pretrain’ with a weighted F1-score of 98.01%. The model that demonstrated the highest performance on the validation dataset, ‘resnest101e’ ranked third in for both weighted and macro-average F1-score, however the performance of this model was consistent with the validation dataset with a less than 1% difference in both weighted and macro-average F1-score between datasets. The ‘resnest101e’ also exhibited a less than 1% difference in accuracy of classification of ‘background’, ‘flower’, ‘leaf’, and ‘branch’ patches for the test dataset compared to the validation dataset. The ‘resnest101e’ was also the top performing model for classification of ‘pod’ patches in the test displaying a 5.06% improvement in accuracy compared to the validation dataset. However, the accuracy of ‘bud’ patch classification for the test dataset using this model was 6.44% lower than for the validation dataset, with a ‘resnest101e_no_pretrain’ model demonstrating the highest accuracy for this class in the test dataset. The top performing models (‘resnest101e_no_pretrain’, ‘resnext101_32x8d’, and ‘resnest101e’) all took 46–48 seconds to complete classification of all patches in the test dataset using a single GPU. Micro- and weighted average F1-score for these models on the testing dataset can be found in **Supplementary Table 7**.

**Table 5:** Performance of 6-label multi-class computer vision classifier models on the test set with 12,461 labelled patches (20% of all manually annotated patches) in descending order according to F1-macro. The F1-score for each class label is also listed. .

Model Name	Rec-macro	Prec-macro	F1-macro	F1-0	F1-1	F1-2	F1-3	F1-4	F1-5	Time
‘resnext101_32x8d’	89.06	**91.85**	**90.28**	99.84	**91.38**	94.47	82.08	**95.86**	78.05	0m 48s
‘resnest101e_no_pretrain’	89.58	90.95	90.08	99.84	91.21	95.21	**84.07**	95.82	74.31	0m 48s
‘resnest101e’	89.02	88.23	88.39	99.83	90.98	**95.33**	69.81	93.13	**81.22**	0m 46s
‘vit_base_patch32_224’	**90.66**	86.24	88.20	**99.85**	90.55	95.30	80.74	93.52	69.21	0m 26s
‘tf_efficientnet_b3_ns’	88.00	87.34	87.61	99.68	89.74	93.60	71.06	93.27	78.29	0m 28s
‘resnet152’	88.96	87.12	87.32	**99.85**	88.76	94.95	84.68	94.40	61.27	0m 32s
‘resnext50d_4s2x40d’	86.12	79.36	81.89	99.76	85.88	93.94	58.06	92.87	60.80	0m 49s
‘swin_base_patch4_window7_224_in22k’	16.67	12.37	14.20	85.20	0.0	0.0	0.0	0.0	0.0	0m 41s

F1-0, ‘Background’; F1-1, ‘Flower’; F1-2, ‘Bud’; F1-3, ‘Leaf’; F1-4, ‘Pod’; and F1-5, ‘Branch’. Time is for model inference on the total 12,461 patch dataset. Bold values indicate the top-performing model for each performance metric specified by column name.

#### 3.2.2 Chain of binary classifier models

The top performing binary classifier model for ‘background,’ ‘flower’, ‘bud’, ‘leaf’, and ‘branch’ classes in the test dataset used a ‘resnext50d_4s2x40d’ architecture, which conformed with binary classifier model performance on the validation dataset (**Table 6**). For ‘pod’ patches a binary classifier with a ‘resnest101e_no_pretrain’ was the top performing model which was also similar to the performance of binary classifier models on the validation dataset. The combined time taken to complete classification of all patches for all classes in the test dataset with the ‘resnext50d_4s2x40d’ model was 4 minutes 52 secs, however with access to a GPU cluster capable of running multiple jobs, binary classification for each class could be run simultaneously reducing the time required to 49 seconds. Weighted average F1-score for these models on the testing dataset can be found in **Supplementary Table 8**.

**Table 6:** Performance of computer vision binary classifier models on the test set with 12,461 labelled patches (20% of all manually annotated patches) in descending order according to F1-macro. The F1-score for each class label is also listed.

Model Name	F1-macro	F1-0	F1-1	F1-2	F1-3	F1-4	F1-5	Time
‘resnext50d_4s2x40d’	**88.76**	**99.88**	**92.36**	**96.75**	**75.80**	**95.97**	71.81	4m 52s(0m 49s)
‘resnest101e_no_pretrain’	87.50	99.85	91.04	95.54	71.34	92.99	**74.21**	4m 38s(0m 46s)
‘resnest101e’	86.90	99.82	89.83	95.98	72.37	92.08	71.34	4m 38s(0m 46s)
‘tf_efficientnet_b3_ns’	86.48	99.85	91.46	95.21	74.26	88.56	69.54	2m 49s(0m 28s)
‘resnet152’	86.40	99.15	90.24	94.69	73.76	89.70	70.87	3m 14s(0m 32s)
‘resnext101_32x8d’	85.41	99.70	89.76	95.28	67.93	90.66	69.14	3m 56s(0m 39s)
‘vit_base_patch32_224’	72.78	99.70	82.87	92.84	24.14	90.58	46.55	2m 27s(0m 25s)
‘swin_base_patch4_window7_224_in22k’	13.96	83.73	0.0	0.0	0.0	0.0	0.0	4m 11s(0m 42s)

F1-0, ‘Background’; F1-1, ‘Branch’; F1-2, ‘Leaf’; F1-3, ‘Bud’; F1-4, ‘Flower’; and F1-5, ‘Pod’. Time is for model inference on the total 12,461 patch dataset. Bold values indicate the top-performing model for each performance metric specified by column name.

#### 3.2.3 Combined plant/background binarization and 5-label multi-class model of plant structures

The top performing 5-label model architecture for classifying *B. napus* plant structures in the test dataset following binary classification of plant patches from background patches was ‘resnext50d_4s2x40d’ with a weighted F1-score of 88.08% when accounting for class imbalance and a macro-average F1-score of 88.12% without accounting for class imbalance (**Table 7**). The ‘resnext50d_4s2x40d’ model architecture performed best as classification of all plant structure classes except ‘leaf’ patches, for which a ‘resnest101e_no_pretrain’ model architecture performed most accurately. Classification of plant patches in the test dataset with the ‘resnext50d_4s2x40d’ model architecture took 14 seconds to complete with a single GPU. After combining the results of binary plant/background classification with 5-label classification of plant structures, the top-performing model architecture was ‘resnext50d_4s2x40d’ with a macro-average F1-score of 90.08% (**Table 8**). Micro- and weighted average F1-score for these models on the training dataset can be found in **Supplementary Tables 9**, **10**.

**Table 7:** Performance of 5-label multi-class computer vision classifier models on the test set with 12,461 labelled patches (20% of all manually annotated patches) in descending order according to F1-macro.

Model Name	Rec-macro	Prec-macro	F1-macro	F1-1	F1-2	F1-3	F1-4	F1-5	Time (m)
‘resnext50d_4s2x40d’	**88.28**	**88.18**	**88.12**	**86.70**	94.00	**82.35**	**89.53**	**88.02**	0m 14s
‘resnest101e_no_pretrain’	86.35	86.79	86.25	84.82	**95.16**	78.88	87.95	84.42	0m 13s
‘resnest101e’	86.44	86.86	86.21	84.85	92.97	80.54	88.84	85.06	0m 13s
‘tf_efficientnet_b3_ns’	85.98	86.55	86.01	83.40	93.60	80.8	88.95	83.30	0m 8s
‘vit_base_patch32_224’	85.90	86.34	85.78	81.99	93.47	79.58	86.87	86.96	0m 7s
‘resnext101_32x8d’	85.58	85.98	85.17	82.93	94.05	74.65	87.26	86.93	0m 12s
‘resnet152’	85.31	86.05	85.07	84.27	94.94	75.07	86.96	84.13	0m 9s
‘swin_base_patch4_window7_224_in22k’	20.00	3.76	6.33	0.0	31.63	0.0	0.0	0.0	0m 11s

F1-0, ‘Background’; F1-1, ‘Branch’; F1-2, ‘Leaf’; F1-3, ‘Bud’; F1-4, ‘Flower’; and F1-5, ‘Pod’. Time is for model inference on the total 12,461 patch dataset.

The F1-score for each class label is also listed. Bold values indicate the top-performing model for each performance metric specified by column name.

**Table 8:** F1 scores for combined results of plant/background classification using the top performing binary classifier model (‘resnext50d_4s2x40d’, see **Table 2**) and 5-label multiclass plant patch classification (**Table 7**) for the test dataset.

Model Name	F1-macro
‘resnext50d_4s2x40d’	**90.08**
‘resnest101e’	88.69
‘resnest101e_no_pretrain’	88.52
‘tf_efficientnet_b3_ns’	88.32
‘vit_base_patch32_224’	88.13
‘resnext101_32x8d’	87.62
‘resnet152’	87.54
‘swin_base_patch4_window7_224_in22k’	21.92

Bold values indicate the top-performing model for each performance metric specified by column name.

### 3.3 Post-processing and application to novel whole-plant images

**Figure 4:**
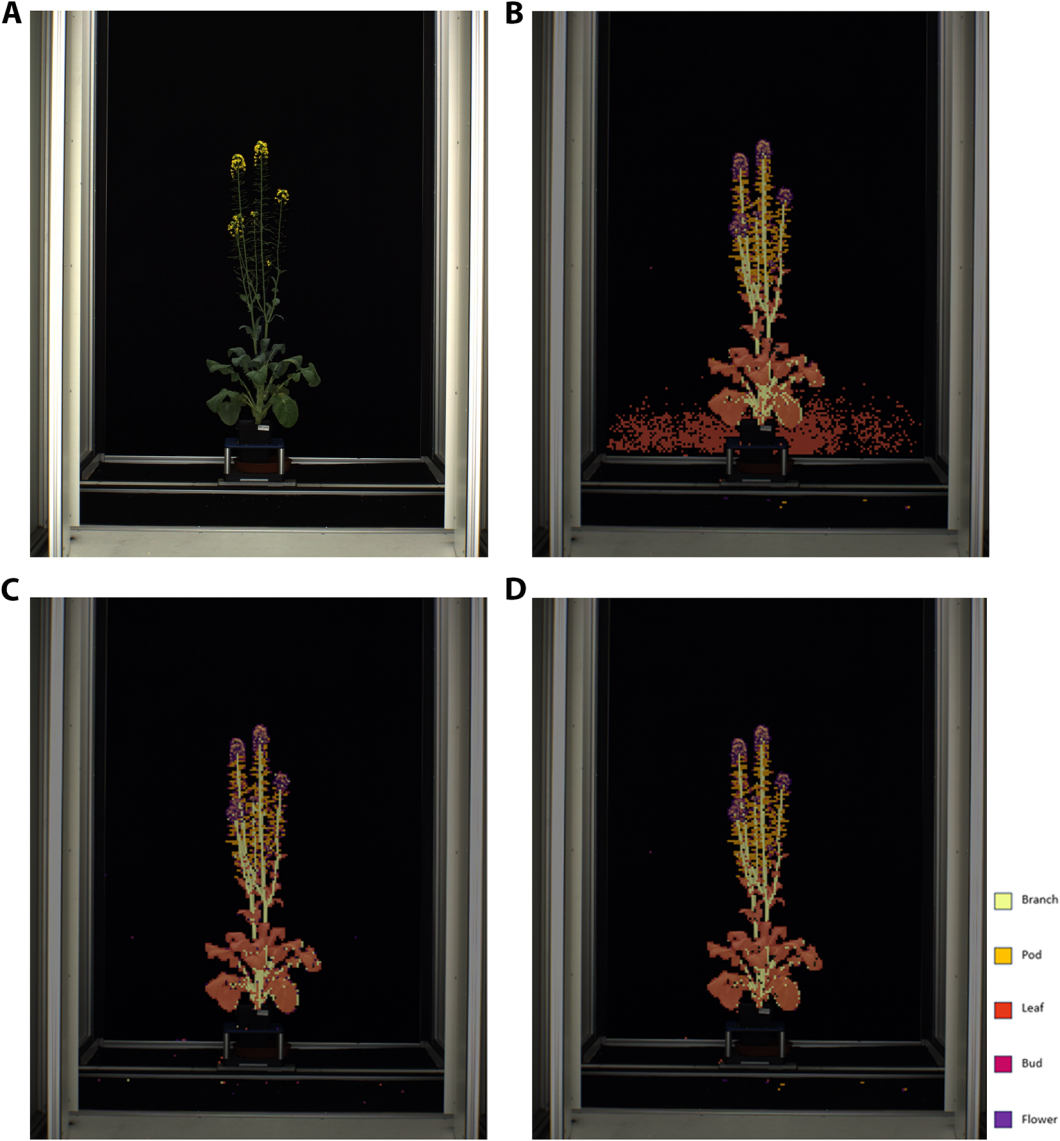
**(A)** Original image of whole *Brassica napus* plant. **(B)** Classification of patches in an image of a whole *Brassica. napus* plant using the top-performing 6-label multi-class classification approach. **(C)** Classification of patches in an image of a whole *Brassica. napus* plant using the top-performing chain of binary classifiers approach. **(D)** Classification of patches in an image of a whole *Brassica. napus* plant using the top-performing combined plant/background binary classification and 5-label plant part classification approach.

Inference was performed on 93 additional images of whole *B. napus* plants from the ‘Collection of side view and top view RGB images of *B. napus* from a large scale, high throughput experiment’ (Williams et al., 2023a) that were not used in model training, validation, or testing datasets. Predictions for each ten-by-ten pixel patch were generated using the top-performing models for each modelling approach (6-label classification, chain of binary classifiers, and combined binary plant/background classification with 5-label plant part classification) and overlaid with the original image in order to visualize and assess model performance and sources of error in classifying novel intact whole plant images. An example of an original inference image can be seen in **Figure 4A**, with classification of patches in this image using the top-performing 6-label shown in **Figure 4B**, the top-performing chain of binary classifiers approach in **Figure 4C**, and top-performing combined binary plant/background with 5-label plant part classification in **Figure 4D**. The results of 6-label classification with the ‘resnest101e’ were applied directly to the novel images without any further preprocessing. When compared to the original image (**Figure 4A**), this approach exhibited substantial misclassification errors where background patches were wrongly identified as ‘leaf’ patches (**Figure 4B**). To get the final classification for each image using the top-performing chain of binary classifiers approach (‘resnext50d_4s2x40d’ binary models for all but pod patches which were classified using the binary ‘resnest101e_no_pretrain’ model) the prediction and confidence generated for each patch by a the binary classifier for each of the six labels was compared so that final label was the label for which the confidence that the patch contained that label was highest. This approach produced similar results to 6-label classification with the most common source of error being background patches misclassified as ‘leaf’ patches (**Figure 4C**; **Supplementary Image 7-12**). Final classification for each image using the combined binary plant/background was achieved by first using the ‘resnext50d_4s2x40d’ binary classifier to predict whether each patch in the image contained the background or part of the plant, and then automatically passing plant patches to the ‘resnext50d_4s2x40d’ 5-label model for to predict which part of the plant (‘branch’, ‘leaf’, ‘bud’, ‘flower’, ‘pod’) was contained in each plant patch. This approach substantially reduced misclassification of background patches as plant parts as was seen in the 6-label classification and chain of binary classifiers approaches (**Figure 4D**).

## 4 Discussion

The modelling approach that produced the top-performing model for classification of patches in the validation and testing datasets was the 6-label multi-class classification approach. This approach yielded the most accurate classification of validation and testing patches when ranked by macro-average F1-score to prioritize classification of minority plant structure classes. However, when the top-performing models from each of the three modelling approaches (6-label classification, chain of binary classifiers, and combined binary plant/background classification with 5-label plant part classification) were used to classify patches in the context of entire images of *B. napus* plants, as opposed to the isolated patches classified in the model validation and testing, the combined binary plant/background classification with 5-label plant part classification was found to be the most accurate (weighted F1-score of 88.08%). The overall top-performing model pipeline was therefore found to be a combination of the binary classifier of plant versus background patches and a 5-label multi-class classification model, both using a ‘resnext50d_4s2x40d’ model architecture.

The success of the combined binary plant/background classification with 5-label multiclass ‘resnext50d_4s2x40d’ model can be attributed to the high accuracy this approach displayed at distinguishing background from plant patches (99.88%). This may be partially due to the fact that the whole plant images were similar to the ‘railspace’ images used in initial development of the MapReader pipeline in that they both contained a high number of patches containing ‘background.’ Misclassification of part of the background as part of the plant was the most common source of error in the inference results of the 6-label multi-class classification. This is likely due to ‘leaf’ patches often being a solid dark green color that was sometimes confused with the solid black background, and ‘bud’ and ‘pod’ patches often containing only a small portion of the relevant plant part on the edge of the patch with the rest of the patch taken up by solid black background. Creating final predictions labels from a combination of all binary classifiers for each class and using the highly accurate binary background/plant model to set labels for ‘background’ patches in the first step of the combined approach eliminated the majority of misclassification errors for these patches. As ‘background’ patches made up the majority of each image, this greatly increased the accuracy and usefulness of predictions for analysis of whole plant images. However, since the combined binary plant/background classification with 5-label plant part classification was more efficient than the chain of binary classifiers approach in terms of both pipeline complexity and time required to complete training and inference, it is recommended that this model be applied to the wider dataset of time-series images of whole *B. napus* plants. Automated passing of patches identified as part of the plant to the 5-label plant part model for further classification ensured this method was more efficient in terms of time and researcher input required to complete model training and inference.

In model validation and testing, classification of ‘bud’ and ‘pod’ patches were found to have the highest error rates (F1-score of 75.80-77.20 and 71.81-76.16 respectively). This is likely due to the fact that ‘bud’ and ‘pod’ patches were the most underrepresented classes, and binary classification models often tend to skew towards categorizing data into the majority class, in this case patches not containing flower buds or pods, leading to lower recall of the minority class (Rawat and Mishra, 2022).

Overall, the combined plant/background binarization and 5-label multi-class plant patch classification models tended to perform better (82.35%) than 6-label multiclass (82.08%) or chain of binary classifier models at classifying these most underrepresented classes; ‘bud’ and ‘pod’ patches (75.80%). However, this enhanced performance on these minority classes was offset by less accurate classification or the other plants classes (‘flower,’ ‘leaf’, and ‘branch’ patches) compared to the other two modelling approaches when applied to the validation and testing datasets (86.70% as opposed to 99%). This may be due to the plant classes appearing more similar to each other without the additional context of ‘background’ patches, leading to greater confusion between plant classes than when this context is retained in model training. When applied to novel whole plant images, the rate of confusion between plant classes was similarly low for all three approaches, providing further support for the benefits of using the combined approach to analyses whole plant images due to the overall low rate of misclassification errors between patches containing different plant parts and between patches containing part of the plant and ‘background’ patches.

Systemically, across all model architectures and modelling approaches, the most common sources of error were ‘bud’ and ‘pod’ patches being misclassified as ‘background’ patches. This was likely due to the small number of labelled patches for these classes to compared to others in the dataset, as well as the fact that both flower buds and pods are small and thin in appearance, and placed at the extremities of the plant, leading to patches for these classes often containing a large amount of background and a small amount of plant material of the relevant class. Overall, there was relatively little confusion between plant classes, which was unexpected as ‘pod,’ ‘branch’ and ‘leaf’ patches can appear very similar to the human eye. This suggests that the difference in shape and coloration of these structures is sufficiently different in *B. napus* plants for computer vision to reliably distinguish between, solidifying this species as a good candidate for automated image analysis as well as highlighting the potential application of these models to other species with similar phenotypic traits such as other Brassica species.

The accuracy of the top-performing models for all three modelling approaches was similar to the top-performing model for classification of railspace in the original development use case for the MapReader modelling framework (Hosseini et al., 2022). This provides strong evidence for the versatility of this framework, both in terms of application to developing models for automated analysis of images from a vast variety of scientific and humanities disciplines, but also in terms of allowing great flexibility in the modelling approaches the MapReader framework can facilitate. Despite being developed originally to compare multi-class modelling approaches, the MapReader framework proved easily adaptable to a chain of binary classifier approach and hierarchical, multi-step pipeline incorporating multiple different modelling approaches (Hosseini et al., 2022). The MapReader annotation tool also played a crucial role in being able to develop a reliable model for the classification of patches in whole *B. napus* plant images, as the functionality provided by the tool to filter patches to easily locate and annotate patches for underrepresented classes greatly reduced the time and researcher effort that would have otherwise been required to label sufficient training data.

The high accuracy of the top-performing combined plant/background binarization and 5-label multi-class model suggests that it can be applied to the full whole *B. napus* plant dataset to quantify the abundance and distribution of plant structures, record when structures first appear, such as first flowering and pod development and track how these change over time throughout the time-series images of each plant. This extracted dynamic phenotype data can then be linked to the data that is available on the genetics, management, and environmental conditions of the imaged plants, allowing for statistical analysis of how these factors impact the processes of plant growth at an individual scale. These insights have the potential to further our understanding of how to breed and manage *B. napus* to produce high oilseed yields under varying climate conditions, as the automated analysis of the time-series images enabled by the model developed in this paper allows researchers to collect finer spatiotemporal phenotype data than has previously been feasible with manual image analysis or conventional phenotyping methods (Campbell et al., 2016; Corcoran et al., 2023b; Costa et al., 2019; Lobell et al., 2011).

To improve the performance of the top-performing model on novel whole *B. napus* plant image data it is recommended that the model be fine-tuned with a larger set of plant class patches from the total ‘Collection of side view and top view RGB images of *B. napus* from a large scale, high throughput experiment’ (Williams et al., 2023a). This can be achieved efficiently by using the top-performing binary classifier model for distinguishing between background and plant patches, which was found to be over 99% accurate, to filter background patches from the patch dataset so that the annotators are presented with only patches containing part of the plant. It is also recommended that the model be applied to the top-down view images of *B. napus*. If the model performance is not found to decrease on application to this dataset, the model can be fine-tuned based on a dataset of labelled patches taken from these images, again using the plant/background binary classification model to speed up the process of collect a representative sample of plant patches. The dynamic phenotype data extracted from the top-down images can then be used to supplement and verify the data extracted from the corresponding side view images, as using multiple views is likely to mitigate the issue of plant parts occluding each other in a single view. If multi-label classification capabilities, wherein multiple plant parts could be labelled within a single patch, were integrated into the MapReader pipeline, it would also be beneficial to explore the use of larger patch sizes that may provide more information about each plant part to improve the accuracy of classification but cannot currently be used due to the limitation of having to provide a single label per patch.

Application of the model to other *B. napus* datasets collected using more simplistic imaging set-ups, such as using a handheld digital camera with a plant on a turntable, is feasible provided the limitations derived from the data used to train the current version of the model are addressed. Firstly, the scale of images within novel datasets would need to be consistent with that of the ‘Collection of side view and top view RGB images of *B. napus* from a large scale, high throughput experiment’ (Williams et al., 2023a)’, and a variety of patch sizes would need to be explored with the MapReader pipeline to find the size which yields the most accurate results. Secondly, it would be recommended that plants be photographed in front of a plain black background to be consistent with the booth used for photographing plants in the current dataset. Thirdly, as the lighting conditions are very consistent across the current training data, it is likely that the model is likely to be less accurate when applied to images that are significantly darker or brighter. This limitation could be addressed by performing data augmentation on images from the wider ‘Collection of side view and top view RGB images of *B. napus* from a large scale, high throughput experiment’ dataset to randomly alter the brightness and contrast of some images to train a version of the model able to better detect parts of *B. napus* under more varied conditions. Application of the top- performing model to images of other plant species should also be explored, as the training effort for future models will be reduced by the capacity to fine-tune the existing model using a relatively small amount of data, and the ability to use the binary plant/background model to aid in creation of new training data.

As the method outlined in this paper is a patch classification model, rather than an object detection or image segmentation model, it is important to note that it does not directly output the discrete number of plant organs within each image. Instead the model provides a label for what plant organ (or part thereof) is contained within each patch, and the location of each patch. This patch-by-patch breakdown of each image could be highly useful as it allows for identification of when plant organs first appear in a time series of plant growth images for each plant as well as exploration of when, where, and why the total amount of certain plant organs may change over time. The length and angle of branches could also be retrieved based on the patch locations, which can also provide answers to questions about the distribution of plant organs in relation to one another, such as whether more flowers occur on main or secondary branches. Patch classification could also be used to speed up manual analysis of plant phenotyping images to provide number of plant organs as it could be used to filter and show only patches containing plant organs to annotators who could then quickly provide a count.

## Data Availability

The datasets presented in this study can be found in online repositories. The names of the repository/repositories and accession number(s) can be found below: https://research.aber.ac.uk/en/datasets/collection-of-side-view-and-top-view-rgb-images-of-brassica-napus.
