# Supplementary figures and images for: Automated dynamic phenotyping of whole oilseed rape (Brassica napus) plants from images collected under controlled conditions
Source: Front Plant Sci. 2025 May 22;16:1443882. doi: 10.3389/fpls.2025.1443882 (PMC12137291; doi:10.3389/fpls.2025.1443882)

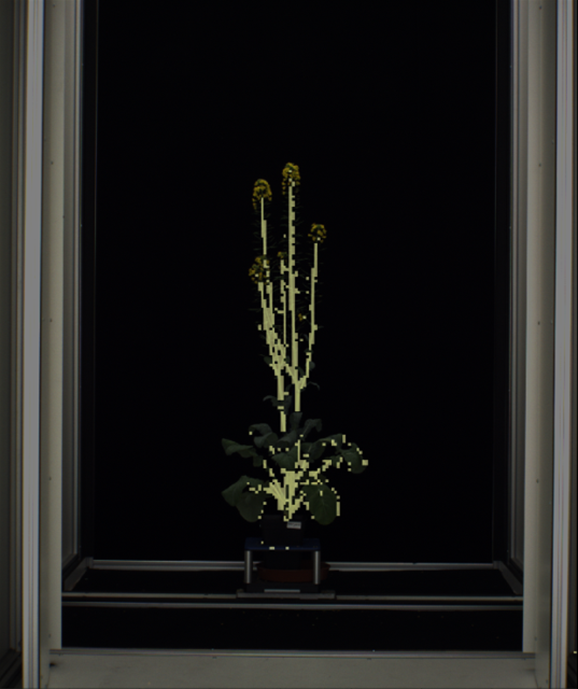

Supplement: Supplementary file 1 [file Image1.tif]

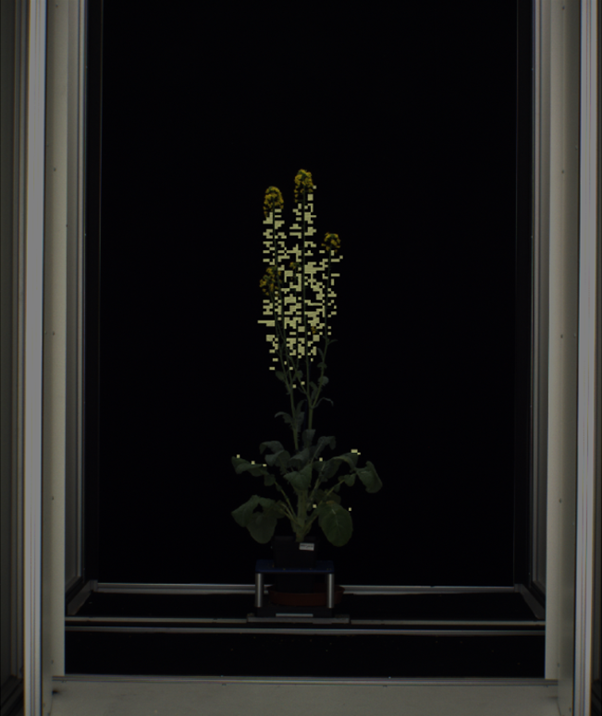

Supplement: Supplementary file 2 [file Image2.tif]

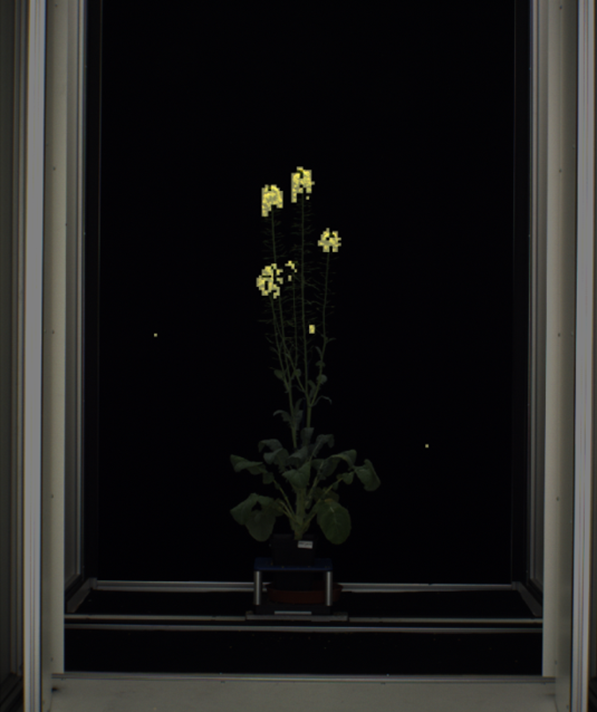

Supplement: Supplementary file 3 [file Image3.tif]

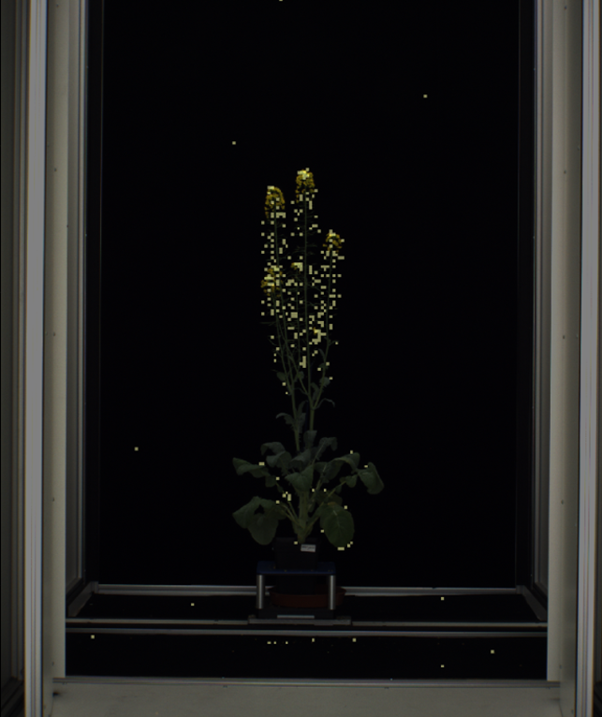

Supplement: Supplementary file 4 [file Image4.tif]

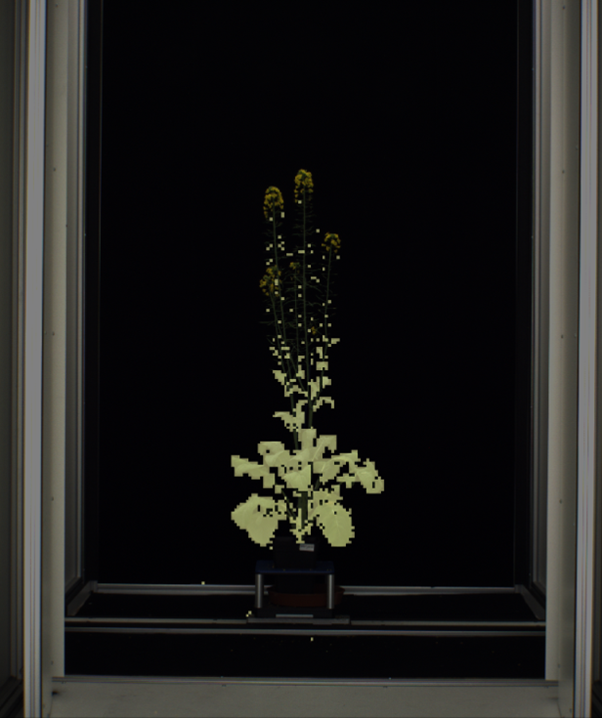

Supplement: Supplementary file 5 [file Image5.tif]

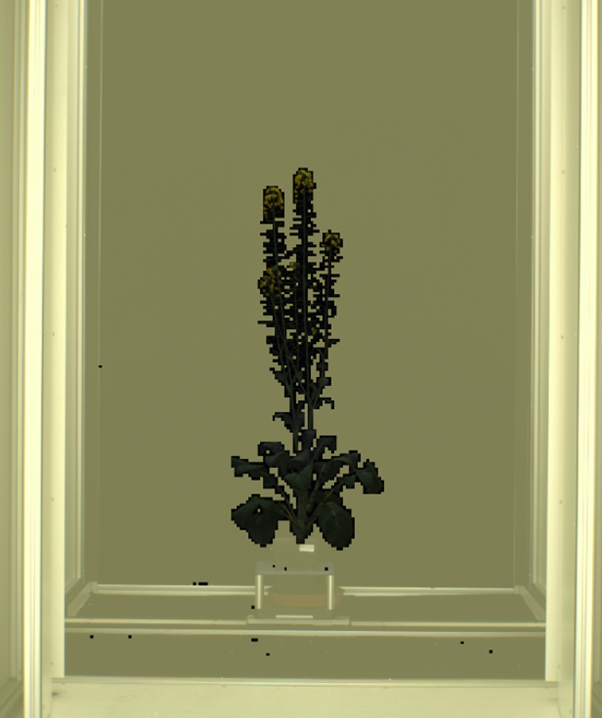

Supplement: Supplementary file 6 [file Image6.tif]

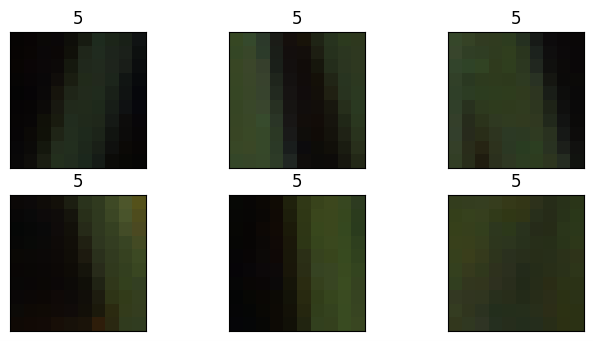

Supplement: Supplementary file 7 [file Image7.tif]

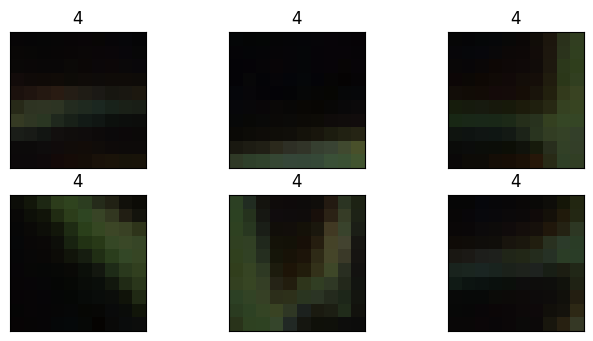

Supplement: Supplementary file 8 [file Image8.tif]

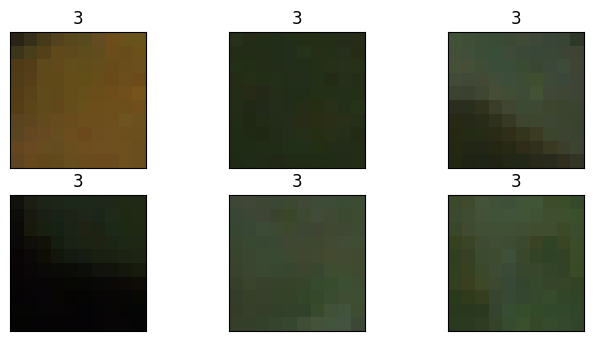

Supplement: Supplementary file 9 [file Image9.tif]

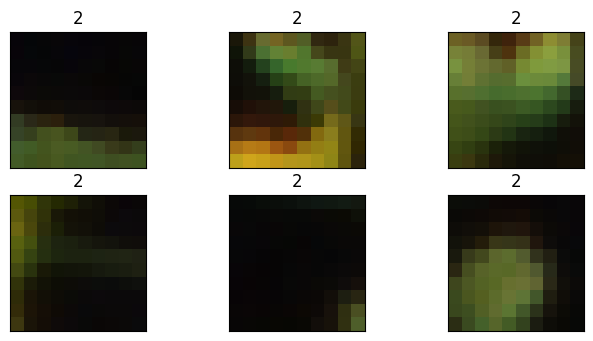

Supplement: Supplementary file 10 [file Image10.tif]

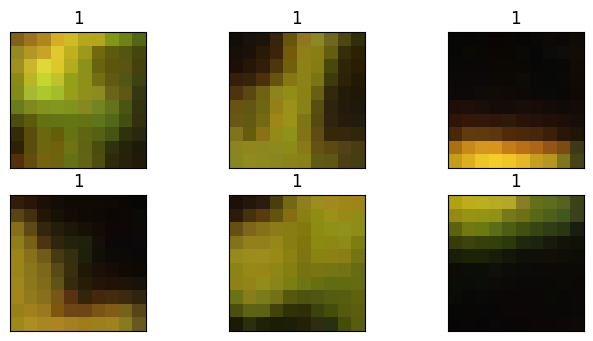

Supplement: Supplementary file 11 [file Image11.tif]

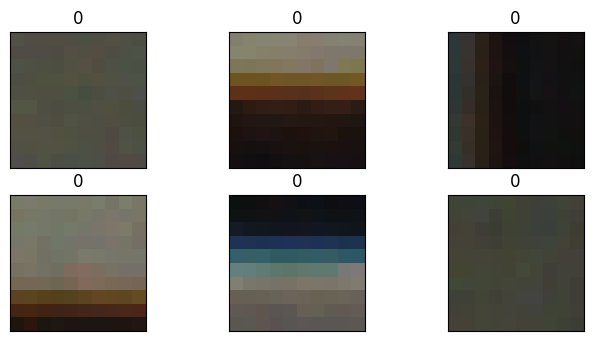

Supplement: Supplementary file 12 [file Image12.tif]
