# Supplementary material for: Automated dynamic phenotyping of whole oilseed rape (Brassica napus) plants from images collected under controlled conditions
Source: Front Plant Sci. 2025 May 22;16:1443882. doi: 10.3389/fpls.2025.1443882 (PMC12137291; doi:10.3389/fpls.2025.1443882)
Supplement: Supplementary file 13 [file Table1.docx]

Supplementary Material

# Supplementary Tables

**Supplementary Table 1. Class distribution of labelled patches from images of whole *Brassica napus* plants in the training, validation, and testing datasets for 6-label multi-class and binary-classifier models.**

| **Class Label** | **Labelled Patches in Training Dataset** | **Labelled Patches in Validation Dataset** | **Labelled Patches in Testing Dataset** | **Total Number of Labelled Patches** |
| --- | --- | --- | --- | --- |
| Background | 26923 | 8975 | 8975 | 44872 |
| Branch | 3255 | 1085 | 1085 | 5426 |
| Leaf | 4765 | 1588 | 1588 | 7942 |
| Bud | 442 | 147 | 147 | 736 |
| Flower | 1493 | 498 | 498 | 2488 |
| Pod | 503 | 168 | 168 | 839 |
| **Total** | **37381** | **12461** | **12461** | **62303** |

**Supplementary Table 2. Class distribution for labelled patches from images of whole *Brassica napus* plants in the training, validation, and testing datasets for the 5-label multi-class plant classification models.**

| **Class Label** | **Labelled Patches in Training Dataset** | **Labelled Patches in Validation Dataset** | **Labelled Patches in Testing Dataset** |
| --- | --- | --- | --- |
| Branch | 3255 | 1085 | 1085 |
| Leaf | 4765 | 1588 | 1588 |
| Bud | 442 | 147 | 147 |
| Flower | 1493 | 498 | 498 |
| Pod | 503 | 168 | 168 |
| **Total** | **10458** | **3486** | **3486** |

**Supplementary Table 3. Performance of 6-label multi-class computer vision classifier models on the validation set with 12,461 labelled patches (20% of all manually annotated patches) including micro-averaged and weighted recalls in descending order according to F1-macro.**

| **Model Name** | **Rec-weight** | **Rec-micro** | **Prec-weight** | **Prec-micro** | **F1-weight** | **F1-micro** |
| --- | --- | --- | --- | --- | --- | --- |
| ‘resnest101e’ | **97.89** | **97.89** | **97.89** | **97.89** | **97.89** | **97.89** |
| ‘vit_base_patch32_224’ | 97.02 | 97.02 | 97.29 | 97.02 | 97.11 | 97.02 |
| ‘resnext101_32x8d’ | 97.13 | 97.13 | 97.09 | 97.13 | 97.10 | 97.12 |
| ‘resnet152’ | 97.22 | 97.22 | 97.22 | 97.22 | 97.19 | 97.22 |
| ‘tf_efficientnet_b3_ns’ | 97.44 | 97.44 | 97.41 | 97.44 | 97.41 | 97.44 |
| ‘resnext50d_4s2x40d’ | 97.59 | 97.59 | 97.59 | 97.59 | 97.57 | 97.59 |
| ‘resnest101e_no_pretrain’ | 97.79 | 97.79 | 97.76 | 97.79 | 97.75 | 97.79 |
| ‘swin_base_patch4_window7_224_in22k’ | 1.18 | 1.18 | 0.01 | 1.18 | 0.03 | 1.18 |

**Supplementary Table 4. Performance of computer vision binary classifier models on the validation set with 12,461 labelled patches (20% of all manually annotated patches) in descending order according to weighted average F1-score.**

| **Model Name** | **F1-weight** |
| --- | --- |
| ‘resnext50d_4s2x40d’ | **98.08** |
| ‘resnest101e_no_pretrain’ | 97.68 |
| ‘resnest101e’ | 97.64 |
| ‘tf_efficientnet_b3_ns’ | 97.64 |
| ‘resnet152’ | 97.02 |
| ‘resnext101_32x8d’ | 97.36 |
| ‘vit_base_patch32_224’ | 95.77 |
| ‘swin_base_patch4_window7_224_in22k’ | 63.25 |

**Supplementary Table 5. Performance of 5-label multi-class computer vision classifier models on the validation set with 12,461 labelled patches (20% of all manually annotated patches) including micro- and weighted-averaged precision, recall and F1-score.**

| **Model Name** | **Rec-weight** | **Rec-micro** | **Prec-weight** | **Prec-micro** | **F1-weight** | **F1-micro** |
| --- | --- | --- | --- | --- | --- | --- |
| ‘resnext50d_4s2x40d’ | 91.77 | 91.77 | 91.90 | 91.77 | 91.79 | 91.77 |
| ‘resnext101_32x8d’ | 91.68 | 91.68 | 91.78 | 91.68 | 91.60 | 90.68 |
| ‘tf_efficientnet_b3_ns’ | 91.57 | 91.57 | 91.91 | 91.57 | 91.69 | 91.57 |
| ‘resnest101e’ | 91.74 | 91.78 | 91.81 | 91.74 | 91.77 | 91.74 |
| ‘vit_base_patch32_224’ | 91.25 | 91.25 | 91.20 | 91.25 | 91.15 | 91.25 |
| ‘resnest101e_no_pretrain’ | **91.88** | **91.88** | **92.02** | **91.88** | **91.91** | **91.88** |
| ‘resnet152’ | 91.11 | 91.11 | 91.54 | 91.11 | 91.21 | 91.11 |
| ‘swin_base_patch4_window7_224_in22k’ | 45.55 | 45.55 | 20.75 | 45.55 | 28.51 | 91.25 |

**Supplementary Table 6. Weighted average F1-scores for combined results of plant/background classification using the top performing binary classifier model (‘resnext50d_4s2x40d’, see Table 4) and 5-label multiclass plant patch classification (Table 5) for the validation dataset.**

| **Model Name** | **F1-weight** |
| --- | --- |
| ‘resnext50d_4s2x40d’ | 97.71 |
| ‘resnext101_32x8d’ | 97.66 |
| ‘tf_efficientnet_b3_ns’ | 97.69 |
| ‘resnest101e’ | 97.72 |
| ‘vit_base_patch32_224’ | 97.53 |
| ‘resnest101e_no_pretrain’ | **97.75** |
| ‘resnet152’ | 97.56 |
| ‘swin_base_patch4_window7_224_in22k’ | 80.05 |

**Supplementary Table 7. Performance of 6-label multi-class computer vision classifier models on the test set with 12,461 labelled patches (20% of all manually annotated patches) including micro-averaged and weighted average F1-score.**

| **Model Name** | **Rec-weight** | **Rec-micro** | **Prec-weight** | **Prec-micro** | **F1-weight** | **F1-micro** |
| --- | --- | --- | --- | --- | --- | --- |
| ‘resnext101_32x8d’ | 97.99 | 97.99 | **98.05** | 97.99 | 97.97 | 97.99 |
| ‘resnest101e_no_pretrain’ | **98.03** | **98.03** | 98.02 | **98.02** | **98.01** | **98.03** |
| ‘resnest101e’ | 97.86 | 97.86 | 97.91 | 97.86 | 97.84 | 97.86 |
| ‘vit_base_patch32_224’ | 97.71 | 97.71 | 97.86 | 97.71 | 97.77 | 97.71 |
| ‘tf_efficientnet_b3_ns’ | 97.34 | 97.34 | 97.36 | 97.34 | 97.34 | 97.34 |
| ‘resnet152’ | 97.46 | 97.46 | 97.75 | 97.46 | 97.53 | 97.46 |
| ‘resnext50d_4s2x40d’ | 96.80 | 96.80 | 97.20 | 96.80 | 96.93 | 96.80 |
| ‘swin_base_patch4_window7_224_in22k’ | 74.22 | 74.22 | 55.08 | 74.22 | 63.23 | 74.22 |

**Supplementary Table 8. Weighted average F1-score for performance of computer vision binary classifier models on the test set with 12,461 labelled patches (20% of all manually annotated patches).**

| **Model Name** | **F1-weight** |
| --- | --- |
| ‘resnext50d_4s2x40d’ | **98.12** |
| ‘resnest101e_no_pretrain’ | 97.68 |
| ‘resnest101e’ | 97.55 |
| ‘tf_efficientnet_b3_ns’ | 97.48 |
| ‘resnet152’ | 96.85 |
| ‘resnext101_32x8d’ | 97.25 |
| ‘vit_base_patch32_224’ | 95.64 |
| ‘swin_base_patch4_window7_224_in22k’ | 60.29 |

**Supplementary Table 9. Performance of 5-label multi-class computer vision classifier models on the test set with 12,461 labelled patches (20% of all manually annotated patches) including micr0-averaged and weighted average F1-score.**

| **Model Name** | **Rec-weight** | **Rec-micro** | **Prec-weight** | **Prec-micro** | **F1-weight** | **F1-micro** |
| --- | --- | --- | --- | --- | --- | --- |
| ‘resnext50d_4s2x40d’ | **88.18** | **88.18** | **88.20** | **88.18** | **88.08** | **88.18** |
| ‘resnest101e_no_pretrain’ | 86.35 | 86.35 | 86.68 | 86.35 | 86.19 | 86.35 |
| ‘resnest101e’ | 86.35 | 86.35 | 86.91 | 86.35 | 86.19 | 86.35 |
| ‘tf_efficientnet_b3_ns’ | 86.03 | 86.03 | 86.48 | 86.03 | 86.00 | 86.03 |
| ‘vit_base_patch32_224’ | 85.95 | 85.95 | 86.37 | 85.95 | 85.82 | 85.95 |
| ‘resnext101_32x8d’ | 85.49 | 85.49 | 85.95 | 85.49 | 85.09 | 85.49 |
| ‘resnet152’ | 85.57 | 85.57 | 86.10 | 85.57 | 85.25 | 85.57 |
| ‘swin_base_patch4_window7_224_in22k’ | 18.78 | 18.78 | 3.53 | 18.78 | 5.94 | 18.78 |

**Supplementary Table 10. Weighted average F1 scores for combined results of plant/background classification using the top performing binary classifier model (‘resnext50d_4s2x40d’, see Table 4) and 5-label multiclass plant patch classification (Table 9) for the test dataset.**

| **Model Name** | **F1-weight** |
| --- | --- |
| ‘resnext50d_4s2x40d’ | **97.22** |
| ‘resnest101e’ | 96.85 |
| ‘resnest101e_no_pretrain’ | 97.07 |
| ‘tf_efficientnet_b3_ns’ | 97.22 |
| ‘vit_base_patch32_224’ | 96.53 |
| ‘resnext101_32x8d’ | 96.71 |
| ‘resnet152’ | 96.91 |
| ‘swin_base_patch4_window7_224_in22k’ | 76.03 |
